# Noninvasive In Vivo Assessment of Cardiac Metabolism in the Healthy and Diabetic Human Heart Using Hyperpolarized ^13^C MRI

**DOI:** 10.1161/CIRCRESAHA.119.316260

**Published:** 2020-02-05

**Authors:** Oliver J. Rider, Andrew Apps, Jack J.J.J. Miller, Justin Y.C. Lau, Andrew J.M. Lewis, Mark A. Peterzan, Michael S. Dodd, Angus Z. Lau, Claire Trumper, Ferdia A. Gallagher, James T. Grist, Kevin M. Brindle, Stefan Neubauer, Damian J. Tyler

**Affiliations:** 1 From the Oxford Centre for Clinical Magnetic Resonance Research, Radcliffe Department of Medicine (O.J.R., A.A., J.J.J.J.M., J.Y.C.L., A.J.M.L., M.A.P., C.T., S.N., D.J.T.), University of Oxford, United Kingdom; 2 Department of Physiology, Anatomy and Genetics (J.J.J.J.M., J.Y.C.L., D.J.T.), University of Oxford, United Kingdom; 3 Department of Physics (J.J.J.J.M.), University of Oxford, United Kingdom; 4 School of Life Sciences, Coventry University, United Kingdom (M.S.D.); 5 Sunnybrook Research Institute, Toronto, Canada (A.Z.L.); 6 Department of Radiology (F.A.G., J.T.G.), University of Cambridge, United Kingdom.; 7 Cancer Research UK Cambridge Institute (K.M.B.), University of Cambridge, United Kingdom.

**Keywords:** diabetes mellitus, diabetic cardiomyopathy, hyperpolarized magnetic resonance spectroscopy, magnetic resonance imaging, metabolism, pyruvate dehydrogenase

## Abstract

**Rationale:**

The recent development of hyperpolarized ^13^C magnetic resonance spectroscopy has made it possible to measure cellular metabolism in vivo, in real time.

**Objective:**

By comparing participants with and without type 2 diabetes mellitus (T2DM), we report the first case-control study to use this technique to record changes in cardiac metabolism in the healthy and diseased human heart.

**Methods and Results:**

Thirteen people with T2DM (glycated hemoglobin, 6.9±1.0%) and 12 age-matched healthy controls underwent assessment of cardiac systolic and diastolic function, myocardial energetics (^31^P-magnetic resonance spectroscopy), and lipid content (^1^H-magnetic resonance spectroscopy) in the fasted state. In a subset (5 T2DM, 5 control), hyperpolarized [1-^13^C]pyruvate magnetic resonance spectra were also acquired and in 5 of these participants (3 T2DM, 2 controls), this was successfully repeated 45 minutes after a 75 g oral glucose challenge. Downstream metabolism of [1-^13^C]pyruvate via PDH (pyruvate dehydrogenase, [^13^C]bicarbonate), lactate dehydrogenase ([1-^13^C]lactate), and alanine transaminase ([1-^13^C]alanine) was assessed. Metabolic flux through cardiac PDH was significantly reduced in the people with T2DM (Fasted: 0.0084±0.0067 [Control] versus 0.0016±0.0014 [T2DM], Fed: 0.0184±0.0109 versus 0.0053±0.0041; *P*=0.013). In addition, a significant increase in metabolic flux through PDH was observed after the oral glucose challenge (*P*<0.001). As is characteristic of diabetes mellitus, impaired myocardial energetics, myocardial lipid content, and diastolic function were also demonstrated in the wider study cohort.

**Conclusions:**

This work represents the first demonstration of the ability of hyperpolarized ^13^C magnetic resonance spectroscopy to noninvasively assess physiological and pathological changes in cardiac metabolism in the human heart. In doing so, we highlight the potential of the technique to detect and quantify metabolic alterations in the setting of cardiovascular disease.

## Abstract 2

Supplemental Digital Content is available in the text.

**In This Issue, see p 705**

**Meet the First Author, see p 706**

Type 2 diabetes mellitus (T2DM), even in the absence of coronary artery disease and hypertension, is associated with a 2 to 5-fold increased risk of heart failure through the development of diabetic cardiomyopathy.^1^ With the rapid global increase in the prevalence of obesity, and with it T2DM, it is very likely that there will be a similar increase in the prevalence of diabetic cardiomyopathy. As a result, there is a pressing need to improve our understanding of the mechanisms by which diabetes mellitus can cause heart failure and to develop noninvasive readouts of the mechanisms which underpin this process.

Several mechanisms have been implicated in the pathogenesis of diabetic cardiomyopathy with changes in myocardial structure, calcium signaling, and metabolism all described in animal models.^2^ As the heart requires a vast amount of ATP to maintain contractile function, it is not surprising that there are functional consequences if metabolism is altered, and in T2DM, metabolic alteration is inherent to the underlying disease process. Although diabetes mellitus is characterized by an apparent abundance of substrate with increased circulating levels of both free fatty acids and glucose, the diabetic myocardium uses almost exclusively free fatty acids for the generation of ATP, and its metabolic flexibility is dramatically reduced.^3^ This arises due to the combination of reduced glucose uptake^4^ and increased fatty acid oxidation,^5^ which mediates an inhibition of PDH (pyruvate dehydrogenase) as described by the Randle cycle,^6^ resulting in a reduced efficiency of ATP production.

As both systole and diastole are ATP consuming processes, this leads to a proposed mechanism whereby reduced glucose oxidation acts, via impaired ATP production, to contribute to the development of diabetic cardiomyopathy, with PDH being the central control point. In line with this, we have recently shown that by pharmacologically increasing PDH flux, and therefore rebalancing glucose utilization, it is possible to reverse the diastolic impairment observable in a rodent model of T2DM.^7^ This highlights the importance of PDH in this process as a potential therapeutic target.

Mechanistic insights into diabetic cardiomyopathy to date have, in general, been gained either in animal models, due to the need for invasive procedures or destructive methods which are not feasible in humans, or using positron emission tomography and magnetic resonance spectroscopy (MRS). Positron emission tomography studies have revealed reductions in glucose uptake^8^ and increases in fatty acid oxidation^9^ while MRS studies have shown elevated myocardial triglyceride content^10^ and impaired myocardial energetics,^11^ confirming to a large extent the findings in animal studies. However, gaining a window on important changes at the level of PDH has not been possible without invasive biopsies, making this impractical to assess as a routine biomarker.

One potential solution to this is ^13^C MRS. This technique allows a direct evaluation of the activity of PDH by measuring the conversion of [1-^13^C]pyruvate into [^13^C]bicarbonate (H^13^CO_3_^−^). However, although this is scientifically attractive, conventional ^13^C MRS suffers from an inherently low sensitivity and low signal-to-noise ratio, making scan times very long, and routine acquisition unfeasible at clinical field strengths. This low sensitivity can be overcome using the recent development of hyperpolarized magnetic resonance (MR) technology, which can amplify the ^13^C MRS signal by over 10 000-fold.^12^ Using hyperpolarized [1-^13^C]pyruvate, physiological changes in PDH flux have been demonstrated in animal models of feeding and fasting.^13–15^ In addition, changes in cardiac substrate selection in a variety of pathological situations have been observed,^16–18^ particularly in diabetes mellitus.^7,14,19,20^

The human applications of this technique are in their infancy, with an initial clinical demonstration in a study of patients with prostate cancer,^21^ and 2 smaller feasibility studies, one in the healthy heart^22^ and another in the healthy brain.^23^ Despite its potential, the assessment of either physiological or pathological changes in metabolic flux using hyperpolarized MRS have not yet been undertaken in the human heart.

As such, the primary aim of the work presented here was to provide the first noninvasive in vivo demonstration that physiological and pathological changes in PDH flux can be detected in the human heart using hyperpolarized [1-^13^C]pyruvate MRS. By also assessing other hallmarks of diabetic heart disease, namely impaired energetics (^31^P-MRS), myocardial steatosis (^1^H-MRS), and diastolic impairment (echocardiography), we further aimed to determine the additional information that the hyperpolarized [1-^13^C]pyruvate technique can provide in the detection of pathological changes in the diabetic heart.

## Methods

The data that support the findings of this study are available from the corresponding author on reasonable request.

### Study Cohort and Study Visit

This research was approved by the National Research Ethics Committee service (13/SW/0108) and conducted in accordance with the declaration of Helsinki and the Caldicott principles. All data collection was undertaken at the Oxford Centre for Clinical Magnetic Resonance at the John Radcliffe Hospital, Oxford, United Kingdom between March 2016 and May 2019. Written informed consent was obtained from all those enrolled. Thirteen people with T2DM, and 12 controls were recruited from local advertisements. All participants were aged >18, participants with T2DM were included if they had a recent glycated hemoglobin between 6 and 9%, no change of oral medications during the previous 3 months and were not on insulin therapy. Subjects with T2DM who were taking the oral antihyperglycemic drug, Metformin, were asked to refrain from taking their medication for 12 hours before the study to minimize any potential effect on cardiac redox state.^24^

All study visits began at 7 am following an overnight fast lasting at least 9 hours. Diastolic function (echocardiography), systolic function (CMR), myocardial steatosis (^1^H-MRS), and myocardial energetics (^31^P-MRS) were all assessed in the fasted state. Additionally, hyperpolarized [1-^13^C]pyruvate MRS was undertaken immediately before and 45 minutes after a standardized oral glucose tolerance test consisting of a 75 g glucose dose (taken in under 5 minutes; Rapilose, Galen Ltd, Craigavon, United Kingdom). All MR scanning was undertaken at 3T (Tim Trio MR system, Siemens Healthineers, Erlangen, Germany).

**Figure 1.**
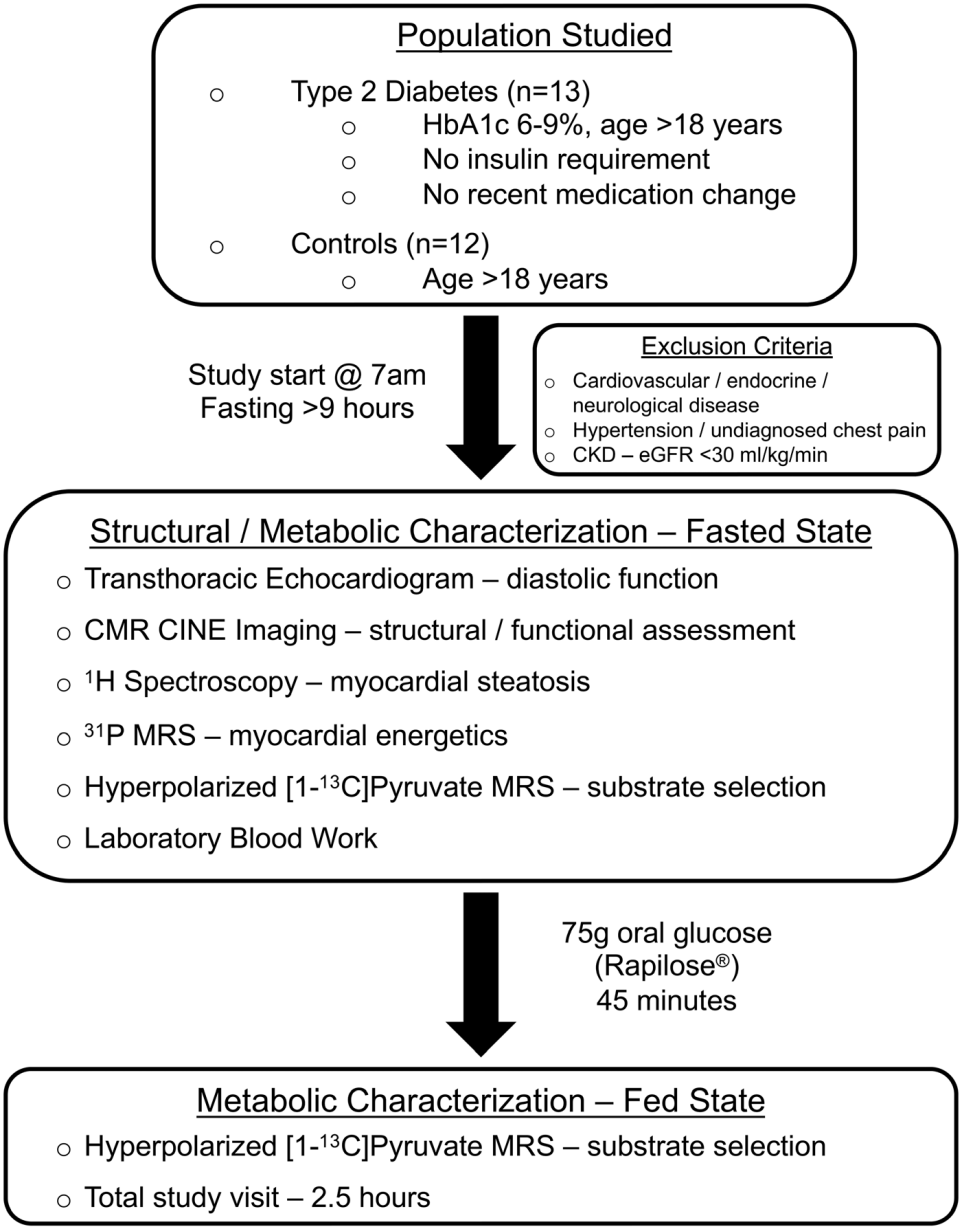
**Outline of our typical study visit.** The fasting stipulation in our study restricted our recruitment to what can be considered a fairly mild phenotype of diabetes mellitus—only those patients receiving oral medication. The total study visit was under 3 hours; however, each hyperpolarized magnetic resonance spectroscopy (MRS) scan took only a few minutes, meaning its addition to the normal length of routine magnetic resonance protocols would be insignificant. CKD indicates chronic kidney disease; CMR, cardiac magnetic resonance; eGFR, estimated glomerular filtration rate; and HbA1c, glycated hemoglobin.

The outline of our study visit is shown in Figure 1, and additional methodological details are given in the Online Data Supplement.

### Dynamic Nuclear Polarization and Production of Hyperpolarized [1-^13^C]Pyruvate

As described in the Online Data Supplement, all starting materials were prepared in a Grade A sterile environment^23^ before being loaded into a General Electric SpinLab system (GE Healthcare, Chicago) for the process of Dynamic Nuclear Polarization.^12^ Sufficient polarization levels were achieved after 2 to 3 hours, after which dissolution was undertaken to produce the final hyperpolarized [1-^13^C]pyruvate solution for injection. Solutions were only released for human injection if the following criteria were met: pH 6.7 to 8.4, temperature 25.0°C to 37.0°C, polarization ≥15%, (pyruvate) 220 to 280 mmol/L, (electron paramagnetic agent) ≤3.0 µmol/L, appearance: clear, colorless solution with no visible particulate matter. Administration of the hyperpolarized pyruvate was undertaken through an 18G venous cannula sited in the left antecubital fossa at a dose of 0.4 mL/kg and at a rate of 5 mL per second.

### Hyperpolarized MR Spectroscopy and Data Processing

Subjects were scanned supine and hyperpolarized ^13^C MR spectra were acquired using a 2 channel transmit, 8 channel surface-receive array (Rapid Biomedical, Rimpar, Germany). Hyperpolarized data were acquired from a mid-ventricular 10 mm axial slice, beginning at the start of the injection, using a pulse-acquire spectroscopy sequence acquired ECG-gated to the R-wave with a single slice-selective excitation every heartbeat and run for 4 minutes after injection. Total integrated metabolite-to-pyruvate ratios, known to linearly correlate with first-order chemical kinetic rate constants, were calculated by summing the first 60 seconds worth of spectral data acquired following the initial appearance of the hyperpolarized pyruvate resonance in the acquired spectra.^25^ Further details are provided in the Online Data Supplement.

### Statistical Analysis

All data were analyzed with the operator blinded to the disease status and metabolic state of the data set. Hyperpolarized data sets, quantified as described above, were analyzed with the *lme4* and the *car* packages in R (v3.6.0, R Foundation for Statistical Computing, Vienna, Austria), with metabolic state and disease status considered as fixed effects, and subject ID considered as a random effect, and an ANOVA table computed. Data were subject to a Shapiro-Wilk normality test, and one outlier corresponding to the [^13^C]bicarbonate to [1-^13^C]pyruvate ratio for an unpaired fasted subject with T2DM with a Z-score of 9.4 was identified (Grubb test *P*=0.003, suggesting that point was an outlier). Data derived from this participant were excluded from subsequent analysis. No evidence of heteroscedasticity was found in the acquired ^13^C data (Levene test, *P*=0.301 for [^13^C]bicarbonate to [1-^13^C]pyruvate ratio, *P*=0.635 for [1-^13^C]lactate to [1-^13^C]pyruvate ratio and *P*=0.751 for [1-^13^C]alanine to [1-^13^C]pyruvate ratio). This fact may reflect the comparatively high signal-to-noise ratio of the acquired spectral data, as it is known that the distribution of metabolite ratios is approximately normally distributed in the high signal-to-noise ratio regime.^26^

Unless otherwise stated, all other analyses were performed in GraphPad Prism (GraphPad Software, San Diego, CA) via simple unpaired unequal-variance *t* tests with the canonical *P*<0.05 threshold for statistical significance. All statistical tests performed are reported in Tables 1 and 2 with the exact *P* values quoted.

**Table 1.**
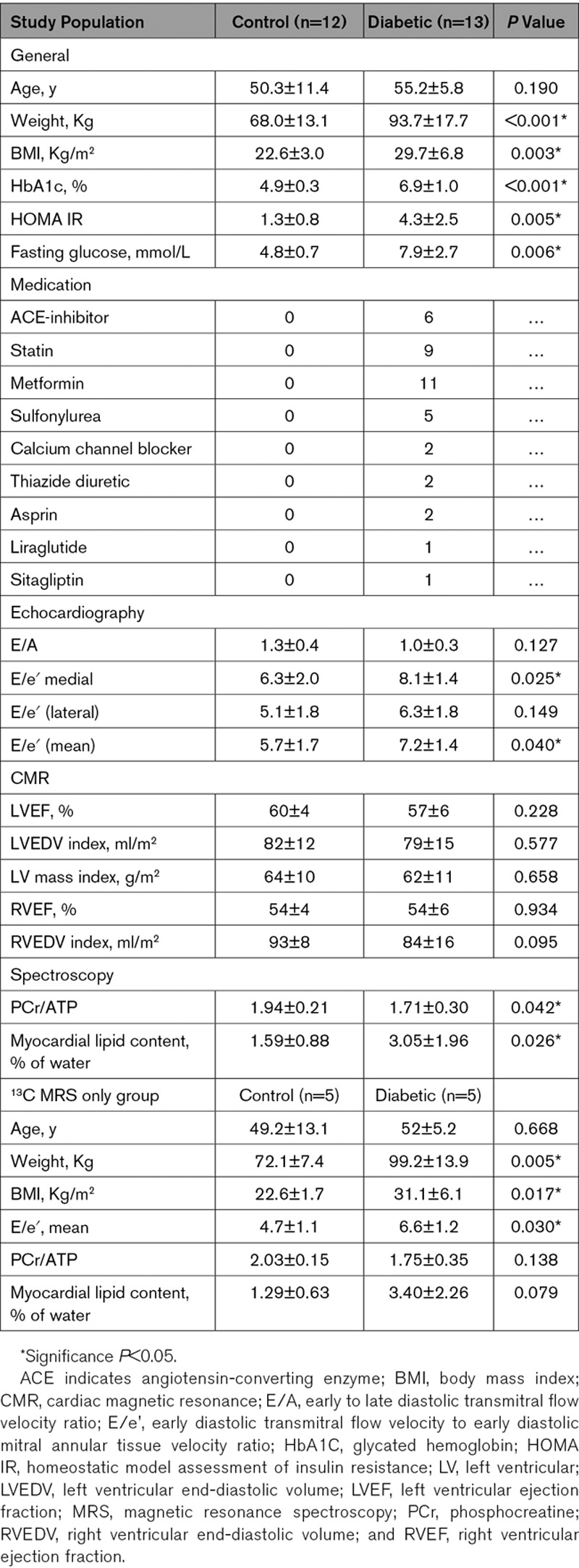
Characteristics of Study Population

**Table 2.**
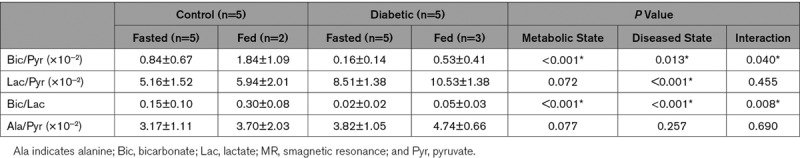
Time-Integrated Metabolite to Substrate Ratios Derived From Hyperpolarized ^13^C MR Data

## Results

### Baseline Population Characterization

**Figure 2.**
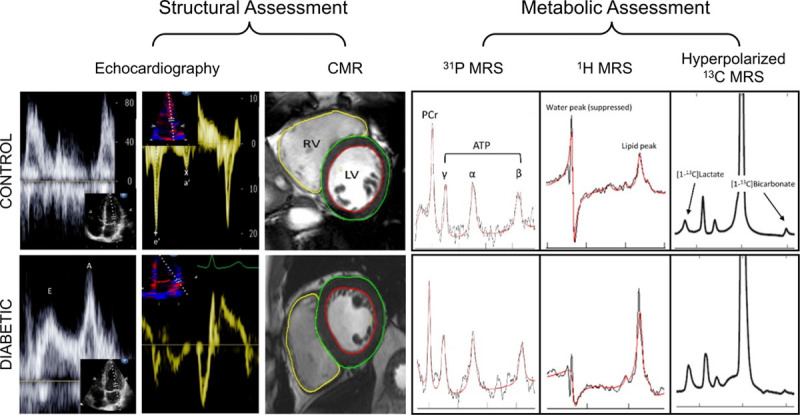
**Example data collected during our study from a recruited control (top row) and a subject with type 2 diabetes mellitus (bottom row).** In characterizing our recruits both structurally (cardiac magnetic resonance [CMR]/Echo) and metabolically (^31^P magnetic resonance spectroscopy [MRS], ^1^H MRS, hyperpolarized ^13^C MRS), we collate the most comprehensive study of the diabetic cardiac phenotype to date. LV indicates left ventricular; and RV, right ventricular.

Healthy controls (n=12) and people with T2DM (n=13) were recruited with no difference in age (controls—50.3±11.4 years, people with T2DM—55.2±5.8 years; *P*=0.190) or sex (controls—8 male/4 female, people with T2DM—11 male, 2 female). Participants with T2DM had significantly higher body mass index than controls (22.6±3.0 versus 29.7±6.8; *P*=0.003), but baseline myocardial structural characteristics assessed by cine-magnetic resonance imaging including left ventricular ejection fraction (60±4% versus 57±6%; *P*=0.228), indexed left ventricular end-diastolic volume (82±12 versus 79±15 mL/m^2^; *P*=0.577) and myocardial mass index (64±10 versus 62±11 g/m^2^; *P*=0.658), were not different between groups (Table 1). Participants with T2DM were confirmed to be more insulin resistant than the controls (homeostatic model assessment of insulin resistance, 1.3±0.8 versus 4.3±2.5; *P*=0.005), with higher fasting blood sugar. Five controls and 5 people with T2DM from within this cohort then went on to have fasting [1-^13^C]pyruvate hyperpolarized MRS, with 5 (2 control, three T2DM) receiving successful repeat [1-^13^C]pyruvate hyperpolarized MRS 45 minutes after glucose ingestion. Again, this smaller hyperpolarized MRS group was well matched for age and myocardial structural characteristics (Table 1). Example data acquired from our study population are shown in Figure 2, demonstrating the breadth of metabolic and structural parameters acquired in a single scanning session.

### Injected Hyperpolarized [1-^13^C]Pyruvate Solution Product Specifications

Hyperpolarized [1-^13^C]pyruvate solution injections were well tolerated by all subjects with no side effects reported. Ten participants (5 controls, 5 T2DM) received a total of 15 injections meeting the release criteria. The quality of these were highly standardized; mean (±SD) pyruvate concentration was 239±8 mmol/L, residual electron paramagnetic agent 1.1±0.7 µmol/L, pH 7.7±0.4, temperature 34±1°C, and polarization 34±13%. The mean polarization time was 150±30 minutes, and dissolution to injection times were all <90 seconds.

### Hyperpolarized ^13^C MRS

**Figure 3.**
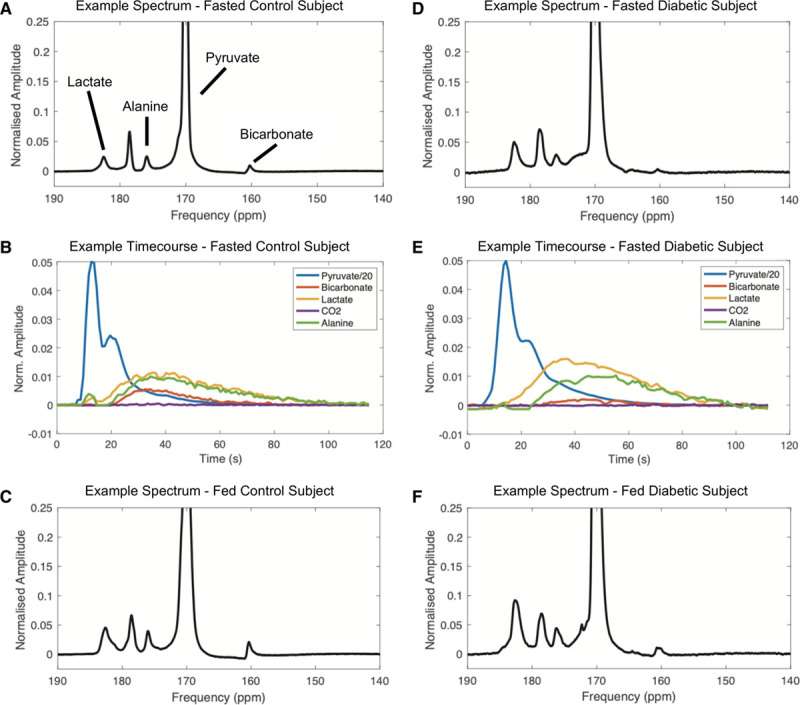
**Representative examples of hyperpolarized magnetic resonance spectra from both a healthy control and a subject with type 2 diabetes mellitus in both the fasted and fed states, with ^13^C containing downstream metabolites labeled.** The [^13^C]bicarbonate resonance is visibly reduced in the subject with type 2 diabetes mellitus with increases seen during feeding in both controls and subjects with type 2 diabetes mellitus. Time courses of the normalized signal amplitudes of downstream ^13^C-labeled metabolic products of administered [1-^13^C]pyruvate (shown in blue), in both a control and a subject with type 2 diabetes mellitus are also shown.

Acquired hyperpolarized spectra were of high quality with peaks corresponding to [^13^C]bicarbonate, ^13^CO_2_, [1-^13^C]lactate and [1-^13^C]alanine (the downstream metabolites of [1-^13^C]pyruvate), clearly visible and appearing 2 to 3 seconds after the ventricular [1-^13^C]pyruvate resonance. Example fed and fasted summed spectra from both a control and subject with T2DM are shown in Figure 3, with typical time courses of substrate and metabolite signal intensities for a control and a subject with T2DM also shown. A summary of time-integrated metabolite to substrate ratios derived from the in vivo hyperpolarized ^13^C MRS data can be found in Table 2.

**Figure 4.**
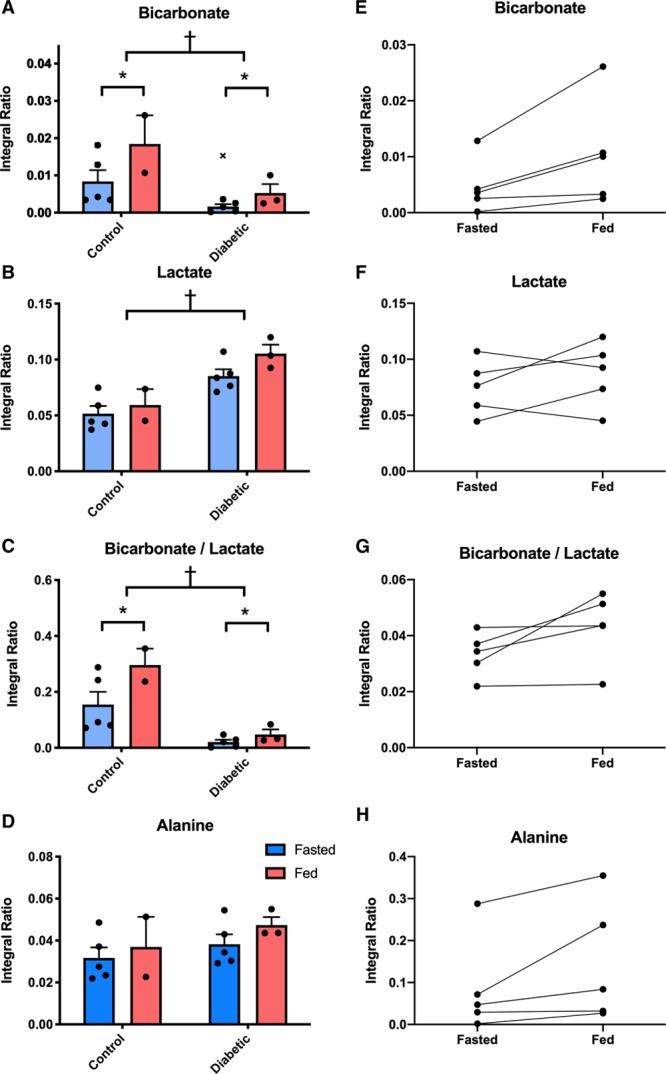
**Plots of metabolic flux data for each metabolic product of administered [1-^13^C]pyruvate.** Flux through PDH (pyruvate dehydrogenase; bicarbonate, **A**) is reduced in the subjects with type 2 diabetes mellitus (*P*=0.013), with increases seen during feeding (*P*<0.001, **E**). Levels of [1-^13^C]lactate were significantly higher in the hearts of people with type 2 diabetes mellitus (*P*<0.001, **B**) with no change observed on feeding (**F**). The ratio of bicarbonate and lactate was significantly lower in the subjects with type 2 diabetes mellitus (*P*<0.001, **C**) and was elevated by feeding (*P*<0.001, **G**). No significant differences in [1-^13^C]alanine were seen across all injections (**D** and **H**). ‘x’ indicates the data point excluded as an outlier. †*P*<0.05 in subjects with type 2 diabetes mellitus vs controls and *P<0.05 in fasted subjects vs fed.

The [^13^C]bicarbonate to [1-^13^C]pyruvate ratio, shown previously to linearly correlate with enzymatic flux through PDH, was significantly reduced by diabetes mellitus (5.3-fold reduction when fasted and 3.5-fold reduction when fed, *P*=0.013). Conversely, the [1-^13^C]lactate to [1-^13^C]pyruvate ratio, reflecting exchange through LDH (lactate dehydrogenase), was increased by diabetes mellitus (1.6-fold increase when fasted and 1.8-fold increase when fed, *P*<0.001). As a marker of the balance between glycolytic and oxidative carbohydrate metabolism,^27^ the ratio of [^13^C]bicarbonate and [1-^13^C]lactate signals showed a significant reduction in relative carbohydrate oxidation in the subjects with T2DM (7.5-fold reduction when fasted and 6-fold reduction when fed, *P*<0.001). Transamination of [1-^13^C]pyruvate to [1-^13^C]alanine was not different between subjects with T2DM and controls (*P*=0.257). Comparisons of enzymatic flux data as assessed by hyperpolarized MRS are summarized in Figure 4.

Hyperpolarized MRS also successfully demonstrated Randle cycle associated increases in PDH flux after feeding with flux significantly increased 45 minutes after the oral administration of 75 g of glucose (*P*<0.001). Importantly, this increase was discernible not only in controls (2.2-fold increase) but also in the subjects with T2DM (3.3-fold increase), in spite of the impaired basal PDH flux we have demonstrated in this condition. There were no statistically significant differences in LDH flux (*P*=0.072) or the rate of pyruvate transamination (*P*=0.077) between the fasted and fed states.

### ^31^P and^1^H MRS

As expected, within the wider study population, diabetes mellitus significantly impaired cardiac diastolic function (mean E/e′ 5.7±1.7 versus 7.2±1.4; *P*=0.040), myocardial energetics (phosphocreatine [PCr]/ATP 1.94±0.21 versus 1.71±0.30; *P*=0.042), and increased myocardial triglyceride content (1.59±0.88 versus 3.05±1.96; *P*=0.026). The effect sizes for these differences (E/e′=0.963, PCr/ATP=0.888, myocardial triglyceride content=0.961, G*Power 3.1) were all lower than the effect sizes calculated for the differences observed between the fasted controls and the subjects with T2DM from the ^13^C enzymatic flux data reported above (bicarbonate/pyruvate=1.405, lactate/pyruvate=2.308, bicarbonate/lactate=1.803). This means that, when comparing 2 groups with a simple Student *t* test, to observe the differences seen here at a *P* value of 0.05 with a power of 90% would require group sizes of 24, 28, and 24 for E/e′, PCr/ATP and myocardial triglyceride content respectively versus group sizes of 12, 6, and 8 for bicarbonate/pyruvate, lactate/pyruvate, and bicarbonate/lactate, respectively (G*Power 3.1).

Weak correlations were observed between the PCr/ATP ratio and the metabolic parameters assessed by hyperpolarized MRS (ie, positive correlations between PCr/ATP and the bicarbonate/pyruvate, alanine/pyruvate and bicarbonate/lactate ratios and a negative correlation between PCr/ATP and the lactate/pyruvate ratio, but these failed to reach statistical significance, Online Figure I).

## Discussion

In the setting of the rapid global increase in T2DM and its relationship with heart failure, increasing our understanding of the metabolic changes that occur in diabetes mellitus is becoming increasingly important. Using a hyperpolarized [1-^13^C]pyruvate tracer, we have shown that, following glucose ingestion, the myocardium increases pyruvate oxidation through PDH (PDH Flux), in line with the metabolic alterations proposed by the Randle cycle.^6^ In addition, we have also shown in patients with T2DM and diastolic dysfunction that PDH flux is reduced, similarly to alterations seen in animal models.^7,20^ This, therefore, represents the first noninvasive demonstration of physiological and pathological changes in PDH flux in the human heart using hyperpolarized MRS. Furthermore, we have used ^31^P and^1^H spectroscopy to confirm that, in the presence of reduced PDH flux, the diabetic myocardium has reduced myocardial energetics (PCr/ATP ratio) and increased myocardial triglyceride content. This is the first human study to use the multinuclear combination of ^1^H, ^31^P, and ^13^C MR spectroscopy to interrogate myocardial metabolism and confirms the potential of hyperpolarized MRS for translation to the clinical quantification of metabolic alterations in cardiac pathology.

### Pyruvate Dehydrogenase Flux

Our demonstration that the fasted heart increases PDH flux after an oral glucose challenge is consistent with the Randle cycle and confirms previous hyperpolarized [1-^13^C]pyruvate experiments in mice,^13^ rats,^14^ and pigs.^15^ While this is an expected result, it is the first demonstration in humans that hyperpolarized [1-^13^C]pyruvate MR can detect physiological changes in myocardial metabolism, an important milestone in its clinical translation.

As the post-glucose scan was undertaken ≈1 hour after the initial fasted scan, there is the possibility that the injected pyruvate dose from the first scan may also have played a part in the increased PDH flux observed. However, it seems unlikely that the ≈1 g dose of pyruvate given would have had a significant impact on top of the 75 g of glucose provided. The variation in PDH flux observed between the fed and fasted states also illustrates that, when considering myocardial metabolic readouts, there is a need to standardize (or at least establish) the prevailing metabolic conditions under which they are made. To date, animal models have used glucose loading before hyperpolarized studies to maximize baseline PDH flux, increasing the power of studies aiming to detect pathological changes.

In contrast to the normal heart, which has metabolic flexibility, the diabetic heart becomes almost exclusively reliant on fatty acids as its main catabolic substrate. This overreliance on fat metabolism is likely underpinned by an impaired ability to uptake glucose and oxidize the resulting pyruvate through PDH. Indeed, animal models of diabetes mellitus have shown PDH inhibition both ex vivo^28^ and in vivo.^14^ In line with this, we have shown here in people with T2DM, that myocardial PDH flux is reduced compared with the normal healthy heart. Minimal discernible flux through PDH was observed in the fasted diabetic state, with only a small increase demonstrated after glucose loading, however, our findings show that hyperpolarized [1-^13^C]pyruvate studies aimed at measuring alterations in PDH flux in patients with T2DM are indeed feasible.

### Linking Altered Substrate Metabolism to Altered Function

As diastole is more susceptible to ATP shortage than systole, alterations in substrate selection may act via reduced efficiency of ATP production initially as diastolic dysfunction, which is an almost universal finding in T2DM.^29,30^ In line with this, we have shown here that the diabetic myocardium has reduced pyruvate oxidation (reflective of reduced glucose utilization), increased triglyceride deposition (suggestive of excess fatty acid uptake), reduced myocardial energetics (with reduced PCr/ATP), and diastolic dysfunction. As the diabetic phenotype in this study was not advanced or severe (we excluded subjects requiring exogenous insulin; average glycated hemoglobin was 6.9%), this highlights the metabolic inflexibility of the cardiomyocyte in the setting of lower grades of insulin resistance, and also the ability of hyperpolarized MR to detect early changes in myocardial metabolism in diabetes mellitus.

### Lactate Dehydrogenase Flux

Incorporation of the ^13^C label into [1-^13^C]lactate in our acquired spectra was significantly higher in subjects with T2DM in both fasted and fed states suggesting raised LDH flux in this group. Although it could be assumed that given [1-^13^C]pyruvate flux through PDH was lower, that LDH flux, and therefore the lactate pool size,^31^ would be reciprocally increased, this interpretation may be too simplistic. Other factors should be considered, for example, it has previously been demonstrated that the antihyperglycemic agent, Metformin, has an effect on cardiac redox state that elevates the observed lactate signal.^24^ To minimize this effect, the subjects with T2DM studied were asked to refrain from taking their Metformin on the day of the study. However, we cannot exclude the possibility that a chronic effect of their Metformin treatment may have contributed to the elevated lactate signal observed.

In addition, the myocardial [1-^13^C]lactate signal following injection of [1-^13^C]pyruvate has proven much more diffuse in hyperpolarized short-axis images of the both the human^22^ and pig heart^32^ with a large contribution from the blood pool. Therefore, [1-^13^C]lactate generated in, and effluxed from, the liver may also be contaminating the cardiac readouts.^33^ As such, we must be cautious in interpreting the exact derivation of the increased lactate signal from nonlocalized spectra. With metabolite imaging now possible in the human heart,^22^ this will aid in the localization of the lactate signal and discern whether or not its origin is myocardial.

### Alanine Aminotransferase Flux

In ex vivo models, the rate of pyruvate transamination has been shown to increase proportionally as pyruvate perfusate concentration increases. Labeled alanine is thus a direct measure of the intracellular availability of labeled pyruvate, and the alanine signal has, therefore, been suggested as an alternative normalization standard (as opposed to the pyruvate signal).^34^ Relative stability of [1-^13^C]alanine signals in our study, and lack of difference between groups, suggests cellular bioavailability of administered [1-^13^C]pyruvate was uniform and not a potential confounder of the variation of enzymatic fluxes seen.

### Wider Translation to Clinical Practice

The technology of dissolution dynamic nuclear polarization is still in its infancy. The first demonstration of clinical translation was published in 2013 using a prototype polarizer located inside a cleanroom to prepare sterile injections for prostate cancer patients.^21^ The SpinLab is the clinical-grade second generation of polarizer suitable for preparing sterile injections outside of a controlled pharmaceutical facility, and currently, 10 sites worldwide are injecting hyperpolarized compounds in early-phase clinical trials. Using this clinical system, we have demonstrated the first step in the clinical translation into cardiovascular disease characterization through the observation of metabolic flux changes in the normal and the diabetic human heart. While technically challenging, leading in part to our work being performed on a comparatively small number of subjects, the large effect size of metabolic dysregulation in disease is such that significant differences in myocardial metabolism, known extensively to exist from several decades of previous animal experimentation, as well as the effects of novel therapies, can be conclusively demonstrated in the human heart. Future studies should build on this proof-of-principle to explore the impact of other cardiovascular diseases, as well as the role that possible confounding factors (such as age, sex, medication use) might have on cardiac metabolism.

As hyperpolarized ^13^C-imaging allows the in vivo visualization of cardiac metabolism, it has major advantages over current noninvasive imaging techniques. Hyperpolarized scans are fast (<2 minutes), have no ionizing radiation, and, due to the ability to simultaneously acquire standard magnetic resonance imaging acquisitions, have the potential to directly assess perfusion, ischemia, viability, and altered substrate selection in the same imaging session. However, the technique does have some limitations. First, the rapid decay of the hyperpolarized signal (ie, the T_1_ of hyperpolarized [1-^13^C]pyruvate in solution has been measured to be 67.3±2.5 s at 3T^35^) leads to the requirement to undertake the hyperpolarization process adjacent to the magnetic resonance imaging system and to inject the hyperpolarized tracer immediately after production. While this offers some technical challenges, the work reported here and by others^21–23^ demonstrates that these challenges, as with short-lived positron emission tomography tracers, can be overcome.

Second, in contrast to positron emission tomography systems, which are capable of measuring picomolar amounts of radiolabeled molecules, hyperpolarized pyruvate scans require injection of the tracer at millimolar concentrations. It has previously been suggested that this supra-physiological dose of pyruvate may impact the metabolic processes that are being assessed. However, preclinical work in animals has shown that similar doses (≈320 mol/kg in previous rat studies versus the ≈140 mol/kg used in this work) leads to maximum plasma pyruvate concentrations of ≈250 µmol/L, equivalent to physiological pyruvate concentrations reached during exercise or with dietary interventions.^34^ In addition, preclinical studies have demonstrated tight correlations between in vivo hyperpolarized MRS measurements of PDH flux and ex vivo measurements of PDH enzyme activity.^34^

While the work described here was undertaken at 3T, there are advantages and disadvantages to undertaking hyperpolarized experiments at different field strengths. Higher field strengths provide increased spectral separation between different metabolites and the subsequent benefits in quantification and selection of different metabolites for spectral imaging that this brings. Alternatively, the longitudinal relaxation times of hyperpolarized agents are generally longer at lower field strengths,^35^ and there is improved B_0_ homogeneity which will improve spectral linewidths. As such, 3T seems a reasonable compromise between these competing factors for such initial proof-of-concept studies.

In conclusion, this study provides the first demonstration of the ability of hyperpolarized pyruvate to noninvasively assess physiological and pathological changes in pyruvate dehydrogenase flux in the human heart. In doing so, we highlight the potential of the technique to assess metabolic alterations in a range of cardiovascular diseases.

## Sources of Funding

This study was funded by a programme grant from the British Heart Foundation (RG/11/9/28921). The authors would also like to acknowledge financial support provided by the British Heart Foundation (BHF) in the form of Clinical Research Training Fellowships, a BHF Intermediate Clinical Research Fellowship and a BHF Senior Research Fellowship, respectively (O.J. Rider: FS/14/54/30946, A. Apps: FS/17/18/32449, A.J.M. Lewis: RE/08/004/23915, M.A. Peterzan: FS/15/80/31803, and D.J. Tyler: FS/14/17/30634). J.J.J.J. Miller and M.S. Dodd would like to acknowledge the financial support provided by Novo Nordisk Postdoctoral Fellowships. J.J.J.J. Miller would also like to acknowledge financial support from Engineering and Physical Sciences Research Council. F.A. Gallagher would like to acknowledge Cancer Research UK (CRUK), the CRUK Cambridge Centre, the Wellcome Trust and the Cambridge Biomedical Research Centre. All authors would also like to acknowledge the support provided by the OXFORD-BHF Centre for Research Excellence (grant RE/13/1/30181) and the National Institute for Health Research Oxford Biomedical Research Centre programme.

## Acknowledgments

We would like to thank Laura Rodden, Katy Crofts, Katy Briggs, Matthew Wilkins, and Claire Church and the Clinical Trials Aseptic Service Unit at the Oxford University Hospitals National Health Services Foundation Trust and Anita Chhabra, Marie-Christine Laurent, Vicky Fernandes, and Matthew Locke from the University of Cambridge for their technical expertise in the preparation of the Sterile Fluid Pathways (SFPs) used in this study.

## Disclosures

F.A. Gallagher has received research support from GE Healthcare. K.M. Brindle holds patents in the field of hyperpolarized magnetic resonance imaging (MRI) relating to the use of imaging media comprising lactate and hyperpolarized [^13^C]pyruvate, ^13^C-MR imaging or spectroscopy of cell death, hyperpolarized lactate as a contrast agent for determination of LDH (lactate dehydrogenase) activity and imaging of ethanol metabolism. In addition, K.M. Brindle has research agreements with GE Healthcare which involve the use of hyperpolarized MRI technology. D.J. Tyler holds a patent relating to the use of hyperpolarized [1-^13^C]pyruvate for the assessment of PDH (pyruvate dehydrogenase) flux and has research agreements with GE Healthcare which involve the use of hyperpolarized MRI technology. The other authors report no conflicts.

## Supplemental Materials

Expanded Materials & Methods

Supplemental Tables I–II

Supplemental Figure I

## Supplementary Material



## Novelty and Significance

### What Is Known?

- The way the heart turns fuels (eg, fats, glucose) into energy, called metabolism, is altered in many types of heart disease.
- However, we have very limited techniques available to allow us to measure metabolism in patients.
