# Supplementary material for: Noninvasive In Vivo Assessment of Cardiac Metabolism in the Healthy and Diabetic Human Heart Using Hyperpolarized 13C MRI
Source: Circ Res. 2020 Feb 5;126(6):725–36. doi: 10.1161/CIRCRESAHA.119.316260 (PMC7077975; doi:10.1161/CIRCRESAHA.119.316260)

Supplemental Material

Expanded Materials & Methods

Recruitment Criteria

All participants were aged >18, participants with diabetes were included if they had a recent HbA1c between 6 and 9% and no change of oral medications during the previous 3 months. Those on insulin therapy were excluded due to a fasting stipulation within the study. Patients with a history of cardiovascular, endocrine or neurological disease, pregnancy, uncontrolled hypertension, chronic renal impairment (estimated glomerular filtration rate < 30 ml/kg/min) or undiagnosed chest pain were excluded, as were those with any standard contraindications to MR scanning.

Echocardiography

Standard 2D Transthoracic echocardiographic (TTE) scanning was undertaken on a portable CX50 console (Philips, Eindhoven, Netherlands), and analyzed offline using Xcelera (Philips, Eindhoven, Netherlands). Diastolic function was assessed according to standard guidelines [36] using mitral Doppler inflow (early E, and atrial A), and peak diastolic mitral annular tissue velocity using tissue Doppler assessment (both medial and lateral, e'). Both E/A and mean E/e' are presented.

Cardiac MR imaging and analysis

A series of long and short axis images were recorded for ventricular volumes, ejection fractions and left ventricular mass analysis using a steady state free precession sequence [37], with a 6-channel flexible torso ^{1}H receive array (Siemens Healthineers, Erlangen, Germany). Imaging was ECG-gated during end-expiratory breath holding. Epicardial and

endocardial borders were manually contoured, with subsequent analysis performed using cmr42© (Circle Cardiovascular Imaging Inc, Calgary, Canada).

Myocardial ^{1}H magnetic resonance protocol

A suitable mid-interventricular septal voxel was initially selected for assessment from long and short axis cardiac localizers. As previously described [38], myocardial lipid content was measured from a series of five water-suppressed, T_1 and B_1 insensitive Stimulated Echo Acquisition Mode (STEAM) spectra (TE 10 ms, TR 2 s, 5 averages), using a 6 channel flexible ^{1}H receive array. These were standardized using a single water-unsuppressed spectrum. All data were analysed in MATLAB (Natick, Massachusetts, USA) using an implementation of the AMARES algorithm [39]. The myocardial lipid content was calculated as a percentage relative to the water signal.

Myocardial ^{31}P magnetic resonance protocol

As previously described in our centre [10,40], to minimize heart-coil distance, subjects were positioned prone on a dual-tuned commercially available $^{31}P/^{1}H$ surface coil (Siemens Healthineers, Erlangen, Germany). Long and short axis ^{1}H localizers were acquired to position the Chemical Shift Imaging (CSI) voxel grid. This was performed to ensure one voxel contained mid-septal myocardium at the mid-ventricular level. Saturation bands were used to minimize signal contamination from skeletal muscle and liver. PCr/ATP was measured non-ECG-gated and free breathing, using a short echo time (0.3ms) 3D acquisition-weighted (matrix size 16 × 8 × 8, FOV 240 × 240 × 200 mm) CSI sequence. Excitation with a shaped pulse was centred at -200Hz relative to PCr (between β and γ ATP) to ensure uniform excitation of all high energy phosphate-containing compounds [40]. Spectra were analysed using the AMARES algorithm implemented in

OXSA [41] with prior knowledge relating to the expected chemical shifts and J-couplings of the phosphorus spectrum and corrected for blood pool contamination and partial saturation [40].

Dynamic Nuclear Polarization and production of hyperpolarized [1-^{13}C]pyruvate

Sterile fluid pathways (SFPs) were assembled in a Grade A sterile environment [23] containing 1.47 g [1-^{13}C]pyruvic acid (Sigma Aldrich, Gillingham, UK) and 15 mM AH111501 (Syncom, Groningen, Netherlands) as the electron paramagnetic agent (EPA). SFPs were loaded into a General Electric SpinLab system (GE Healthcare, Chicago, USA) which was used for the process of Dynamic Nuclear Polarization [42]. Sufficient polarization levels were achieved after 2-3 hours. Dissolution was undertaken using 38.5 g of sterile water heated to 130°C under pressure, released through the pyruvate-containing vial into a receiver vessel containing 17.7 g of trometamol buffer solution (600 mM NaOH, 333 mM Tris base, and 333 mg/L disodium EDTA [as the chelating agent], Royal Free Hospital, London, UK) and a further 19.5 g of sterile water. The EPA was removed by filtration prior to the receiver vessel, with the final product for injection drawn from the receiver vessel into a 50 ml injection syringe (Bayer, Indianola, USA) via a further 0.2 µm sterilization filter (Saint-Gobain, Gaithersburg, USA). Rigorous quality control (QC) of the final filtered sodium [1-^{13}C]pyruvate solution was undertaken prior to human injection. This consisted of both online measurements (pyruvate concentration, residual EPA concentration, temperature, polarization, volume) directly from the SpinLab inbuilt QC console, with further 'offline' pH measurement (RQflex 10, Merck, Darmstadt, Germany) and visual inspection of the product (for visible particulates and appearance) undertaken manually prior to release. Pathways were only released for human injection if the following criteria were met: pH 6.7-8.4, temperature 25.0-37.0°C,

polarization ≥ 15%, [pyruvate] 220-280 mM, [EPA] ≤ 3.0 μM, appearance: clear, colourless solution with no visible particulate matter. Pathways not meeting these release criteria were rejected. Hyperpolarized [1-^{13}C]pyruvate solution was administered through an 18G venous cannula sited in the left antecubital fossa, at a dose of 0.4 ml/kg, followed by a 25 ml 0.9% normal saline flush. Injections were performed at a rate of 5 ml per second using a MEDRAD® power injector system (Bayer, Berlin, Germany).

Hyperpolarized MR spectroscopy and data processing

Subjects were scanned supine and short axis localizers, used to plan hyperpolarized data acquisition, were acquired using the inbuilt ^{1}H body coil. ^{13}C MR spectra were acquired using a 2 channel transmit, 8 channel surface receive array (Rapid Biomedical, Rimpar, Germany). A [^{13}C]urea fiducial marker strapped on top of the coil was used to calibrate the ^{13}C centre frequency. Spectra acquired at thermal equilibrium prior to hyperpolarized injection were used to subtract away the fiducial and background thermal-equilibrium fat signal from the hyperpolarized spectra. Hyperpolarized data were acquired from a mid-ventricular 10 mm axial slice, beginning at the start of the injection. To minimise motion, subjects were invited to initiate an end-inspirational breath-hold at the start of the injection, breathing when required after approximately 30 s, before repeating this procedure until the hyperpolarized signal had returned to thermal equilibrium. The MR acquisition was a pulse-acquire spectroscopy sequence with slice selection achieved with a 1.3 ms sinc excitation triggered by ECG-gating to the R-wave. A single excitation and spectroscopic readout was acquired every heartbeat and run for up to four minutes after injection (flip angle 10°, bandwidth 5 kHz, TR 500 ms, 2048 complex points). Multi-coil data were recombined in MATLAB using the Whitened Singular Value Decomposition

algorithm [43], with coil combination weights calculated for spectra with the highest SNR subsequently applied to the entire dataset. Spectra were background-subtracted prior to quantification with the AMARES algorithm [39], with appropriate prior knowledge. Total integrated metabolite-to-pyruvate ratios, known to linearly correlate with first-order chemical kinetic rate constants [44], were calculated from 60 seconds of data taken after the initial appearance of the pyruvate resonance in the spectrum.

Statistical analysis

Linear mixed effects models form a statistically powerful generalisation of traditional ANOVA methods that are effectively able to use repeated measurements even in the presence of missing data, and are comparatively robust [45]. Accordingly, hyperpolarized datasets, quantified as described above, were analysed with the *lme4* [46] and the *car* packages in R (v3.6.0, R Foundation for Statistical Computing, Vienna, Austria), with metabolic state and disease status considered as fixed effects, and subject ID considered as a random effect, and an ANOVA table computed. Data were subject to a Shapiro-Wilk normality test and one outlier corresponding to an unpaired fasted subject with diabetes with a Z-score of 9.4, was identified (Grubb's test $p=.003$, suggesting that point was an outlier) [47]. Data derived from this patient were excluded for subsequent analysis. Unless otherwise stated, all other analyses were performed in GraphPad Prism (GraphPad Software, San Diego, California, USA) via simple unpaired unequal-variance *t*-tests with the canonical $p<0.05$ threshold for statistical significance.

Supplemental Table I: Subject Characteristics

Control Subjects				Diabetic Subjects			
ID	Height (m)	Weight (Kg)	BMI (Kg/m^2)	ID	Height (m)	Weight (Kg)	BMI (Kg/m^2)
1	1.63	52.0	19.57	1	1.79	87.0	27.15
2	1.86	83.5	24.14	2	1.87	115.0	32.89
3	1.60	63.0	24.61	3	1.77	79.0	25.22
4	1.84	70.0	20.68	4	1.74	81.5	26.92
5	1.67	60.0	21.51	5	1.67	112.0	40.16
6	1.68	52.0	18.42	6	1.98	120.0	30.61
7	1.69	75.0	26.26	7	1.63	122.0	45.92
8	1.73	68.0	22.72	8	1.89	86.0	24.08
9	1.83	72.0	21.50	9	1.76	97.0	31.31
10	1.80	95.0	29.32	10	1.79	68.0	21.22
11	1.81	72.0	21.98	11	1.78	78.0	24.62
12	1.59	53.0	20.96	12	1.72	87.0	29.41
				13	1.78	86.0	27.14
Average	1.73	68.0	22.64	Average	1.78	93.7	29.74
St. Dev.	0.10	13.1	3.03	St. Dev.	0.09	17.7	6.81

Control Subjects				Diabetic Subjects			
ID	Age	Sex	Ethnicity	ID	Age	Sex	Ethnicity
1	58	Male	Caucasian	1	51	Male	Caucasian
2	56	Male	Caucasian	2	49	Male	Caucasian
3	56	Male	Caucasian	3	60	Male	Caucasian
4	32	Male	Caucasian	4	50	Male	South Asian
5	36	Female	Caucasian	5	52	Female	Caucasian
6	59	Female	Caucasian	6	58	Male	Caucasian
7	48	Female	Caucasian	7	53	Female	Caucasian
8	64	Male	Caucasian	8	60	Male	Caucasian
9	63	Male	Caucasian	9	53	Male	Caucasian
10	37	Male	Caucasian	10	64	Male	Caucasian
11	39	Male	Caucasian	11	57	Male	Caucasian
12	56	Female	Caucasian	12	64	Male	Caucasian
				13	46	Male	Caucasian

Supplemental Table II: Data for Excluded Subject

PARAMETER	CONTROL (AVERAGE ± STDEV)	DIABETIC (AVERAGE ± STDEV)	EXCLUDED SUBJECT
Age (years)	50.3 ± 11.4	55.2 ± 5.8	50
HbA1c (%)	4.9 ± 0.3	6.9 ± 1.0	7
HOMA IR	1.3 ± 0.8	4.3 ± 2.5	4.2
Fasting Glucose (mmol/l)	4.8 ± 0.7	7.9 ± 2.7	9.1
BMI (Kg/m^2)	22.6 ± 3.0	29.7 ± 6.8	26.9
LVEF (%)	60 ± 4	57 ± 6	57
LV Mass Index (g/m^2)	64 ± 10	62 ± 11	63
E/e' (mean)	5.7 ± 1.7	7.2 ± 1.4	7.9
PCr/ATP	1.94 ± 0.21	1.71 ± 0.30	1.51
Myocardial Lipid Content (% of water)	1.59 ± 0.88	3.05 ± 1.96	3.64
Fasted Bic/Pyr (x10^{-2})	0.84 ± 0.67	0.16 ± 0.14	1.52
Fasted Lac/Pyr (x10^{-2})	5.16 ± 1.52	8.51 ± 1.38	7.16
Fasted Ala/Pyr (x10^{-2})	3.17 ± 1.11	3.82 ± 1.05	3.92

Supplemental Figure I

Correlations between the measured PCr/ATP ratio and the metabolic data acquired (bicarbonate/pyruvate, lactate/pyruvate, alanine/pyruvate and bicarbonate/lactate).

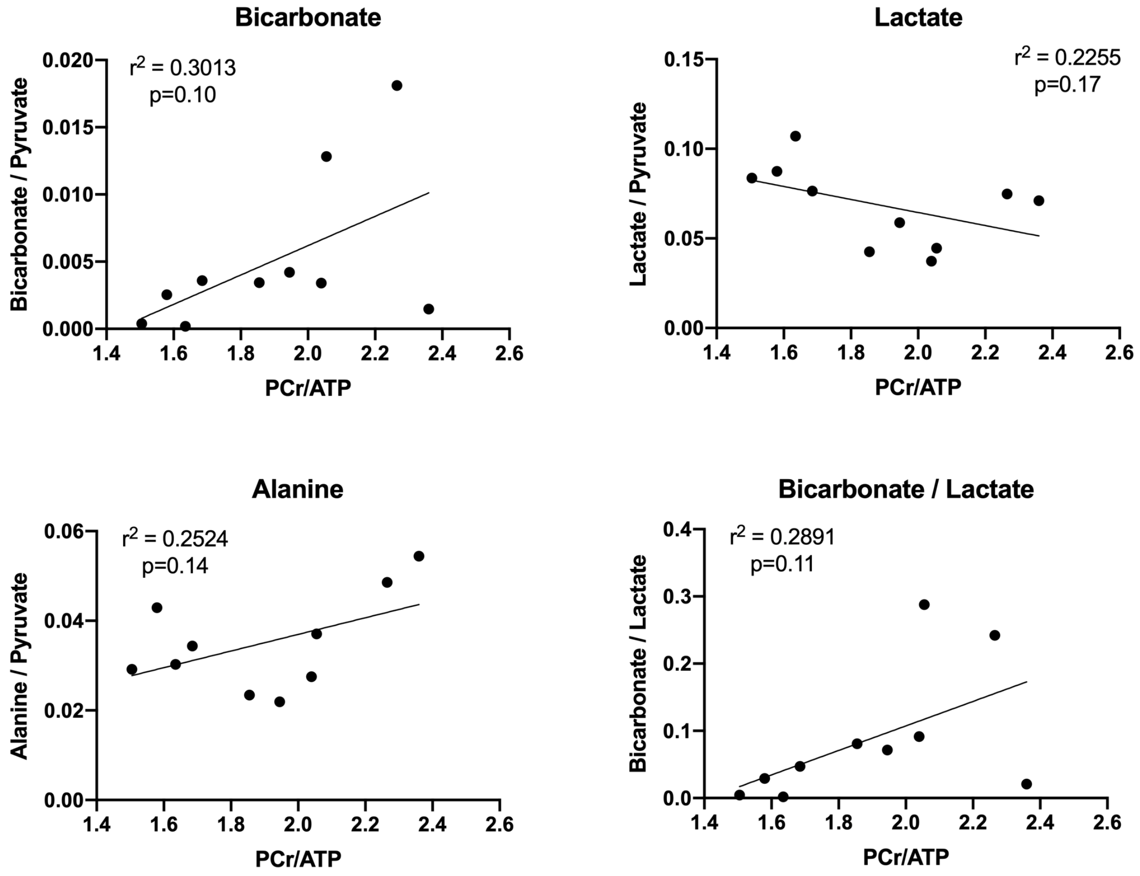

Supplement: Supplementary file 1 [file res-126-725-s001.pdf]
