# Supplementary figures and images for: Noninvasive In Vivo Assessment of Cardiac Metabolism in the Healthy and Diabetic Human Heart Using Hyperpolarized 13C MRI
Source: Circ Res. 2020 Feb 5;126(6):725–36. doi: 10.1161/CIRCRESAHA.119.316260 (PMC7077975; doi:10.1161/CIRCRESAHA.119.316260)

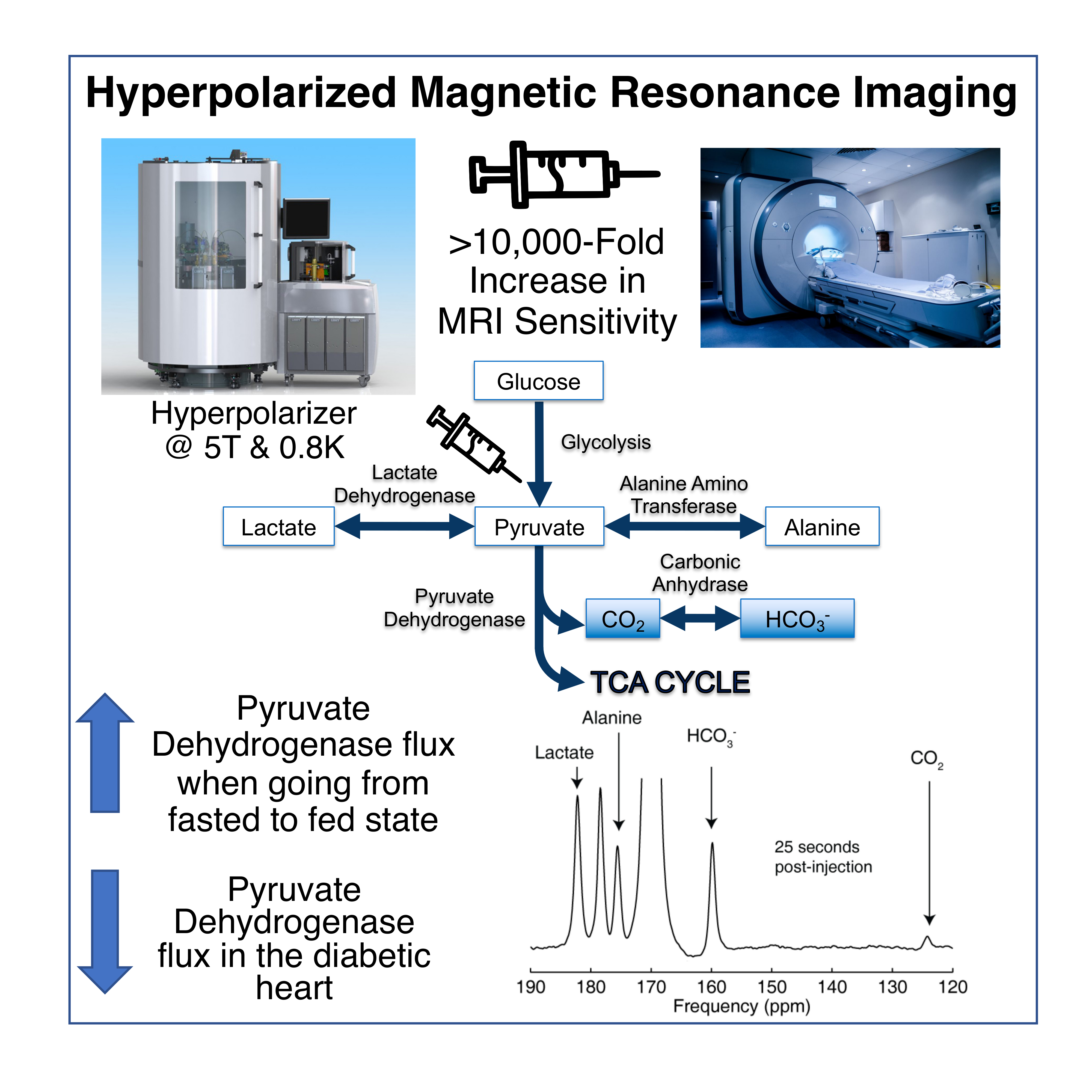

Supplement: Supplementary file 2 [file res-126-725-s002.jpg]
